# Induction of HOXA9 expression in three-dimensional organotypic culture of the Claudin-low breast cancer cells

**DOI:** 10.18632/oncotarget.10491

**Published:** 2016-07-08

**Authors:** Miao Li, Xi Li, Yan Zhuang, Yan Wang, Matthew E. Burow, Bridgette Collins-Burow, Min Xue, Chengjie Song, Bin Shan

**Affiliations:** ^1^ Department of Microbiology and Parasitology, College of Basic Medical Sciences, China Medical University, Shenyang, China; ^2^ Department of Sports Medicine and Joint Surgery, The People's Hospital of Liaoning Province, Shenyang, China; ^3^ Department of Medicine, Tulane University School of Medicine, New Orleans, LA, USA; ^4^ Department of Biological Engineering, Zunyi Medical College Zhuhai Campus, Zhuhai, China; ^5^ Department of Physiology, Xuzhou Medical College, Xuzhou, China; ^6^ Department of Biomedical Sciences, Elson S. Floyd College of Medicine, Washington State University Spokane, Spokane, WA, USA

**Keywords:** breast cancer, extracellular matrix, three-dimensional organotypic culture, gene expression, homeobox gene

## Abstract

The gene expression signatures of the molecular intrinsic subtypes of breast cancer are regulated by epigenetic mechanisms such as methylation of CpG islands in gene promoters. Epigenetic codes can be regulated by the tumor microenvironment. The Claudin-low subtype is associated with triple-negative invasive ductal carcinomas in patients. Herein we explored epigenetic regulation of gene expression in the Claudin-low breast cancer cells by extracellular matrix (ECM), a key component of the tumor microenvironment. We modeled attachment to ECM using laminin rich ECM three-dimensional organotypic culture (lrECM 3D). In 2D and lrECM 3D cultures we examined expression of the homeobox (HOX) genes that epigenetically regulated in development and cancer. We demonstrated induction of the selected HOX genes in lrECM 3D culture of the Claudin-low breast cancer cells MDA-MB-231 and Hs578T. In particular activation of HOXA9 expression in lrECM 3D culture required binding of bromodomain containing 4 to the HOXA9 promoter and involved CpG hypomethylation. Our findings warrant further investigation of the ECM-regulated epigenetic coding of gene expression in the Claudin-low breast cancer.

## INTRODUCTION

The commonly used pathological classifiers in breast cancer are estrogen receptor (ER), progesterone receptor (PR), and human epidermal growth factor receptor 2 (HER2) [1]. Gene expression profiling clusters breast cancer into the following “intrinsic subtypes”: Luminal A, Luminal B, Basal, Claudin-low, and HER2-enriched [1–3]. The gene signatures of these intrinsic subtypes complement the classical pathological markers of breast cancer [4]. These intrinsic subtypes are also observed in human breast cancer cell lines [5]. The Claudin-low subtype mostly correlates with triple-negative (ER-negative, PR-negative, and HER2-negative) invasive ductal carcinomas [5, 6]. Interestingly the Claudin-low subtype is enriched with the biological traits linked to signaling components of the cellular responses to extracellular matrix (ECM) [6–11].

The gene expression signatures of the intrinsic subtypes of breast cancer are governed in part by epigenetic mechanisms [10, 12]. Two major modes of epigenetic inactivation are cytosine methylation of CpG islands by DNA methyltransferease (DNMT) and trimethylation of histone H3 at lysine 27 (H3K27me3) by polycomb repressive complex 2 (PRC2) in gene promoters [13, 14]. Among the DNMT- and PRC2-repressed genes the homeobox (HOX) genes play a critical role in mammary development and breast cancer [15, 16]. Recent advances highlight the importance of the readers of histone modifications. For instance bromodomain containing 4 (BRD4) binds acetylated histones and recruits positive transcription elongation factor-b to promote expression of oncogenes [17, 18].

The tumor microenvironment modulates tumorigenesis extrinsically via ECM [19]. Laminin rich ECM (Matrigel) three-dimensional organotypic culture (lrECM 3D) faithfully recapitulates salient *in vivo* properties of breast cancer cells [20]. Gene expression signatures of various breast cancer cell lines in lrECM 3D culture are tightly correlated with their distinctive morphogenesis, invasiveness, and metastatic properties [21]. Moreover the gene expression signatures from lrECM 3D culture of breast cancer cells hold prognostic values for patients with breast cancer [22].

Emerging evidence from lrECM 3D culture hints regulation of epigenetic codes by ECM [23, 24]. However ECM signaling and epigenetic coding have not been examined in an integrated manner in the Claudin-low breast cancer cells [6, 11]. Herein we aimed to dissect epigenetic regulation of the HOX gene expression by ECM in the Claudin-low breast cancer cells using lrECM 3D culture.

## RESULTS

### Induction of the HOX genes in lrECM 3D culture of the Claudin-low breast cancer cells

**Figure 1:**
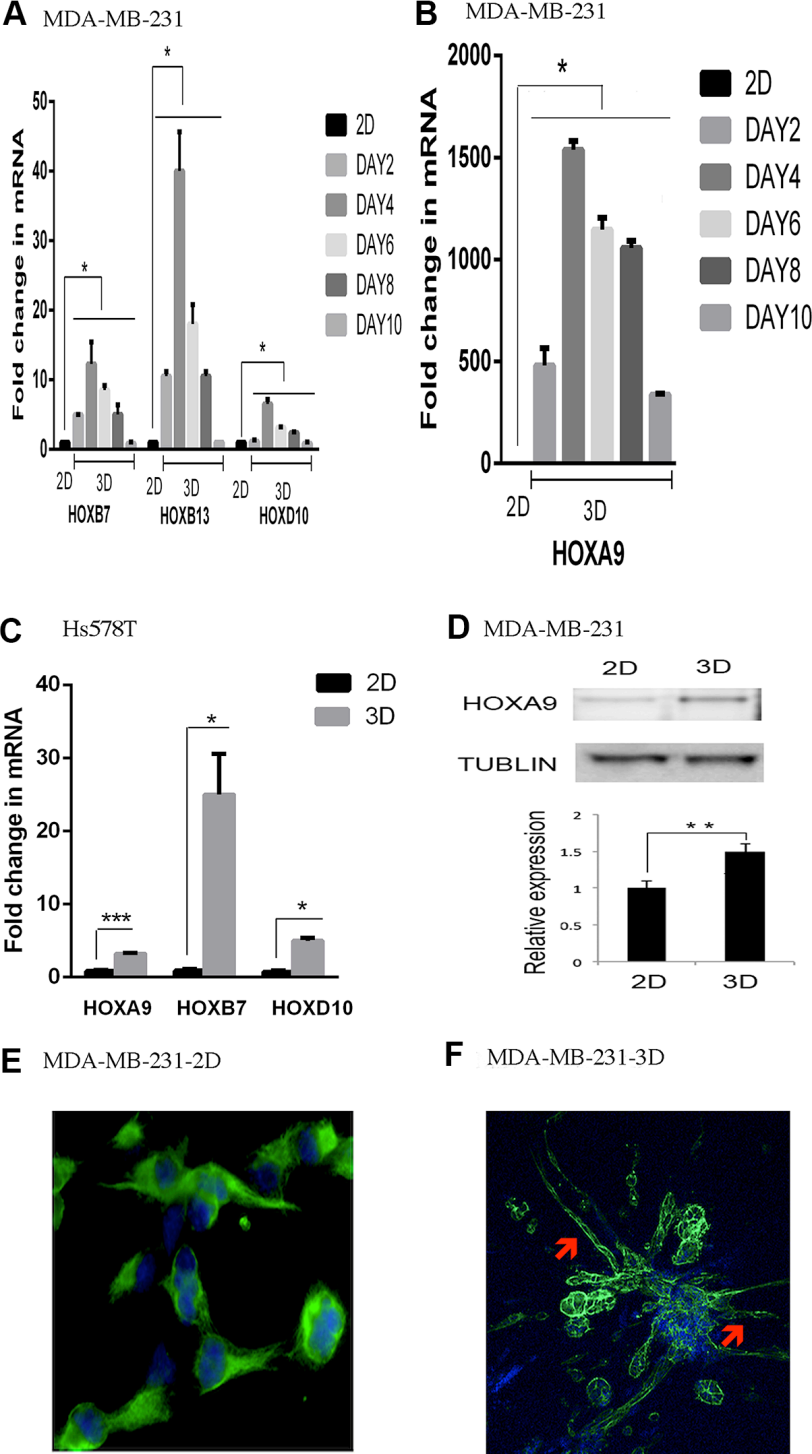
Induction of the HOX genes in lrECM 3D culture (**A**) Total cell RNA was extracted from MDA-MB-231 cells in 2D (day 3) and lrECM 3D cultures at the indicated time points. The mRNA levels of the selected HOX genes were compared between 2D and lrECM 3D cultures using qRT-PCR. A fold change of each transcript at each time point in lrECM 3D culture over 2D culture was obtained by normalizing to the housekeeping gene RPLP0 and setting the values from 2D culture to one. (**B**) Similar to part A except that the mRNA levels of HOXA9 were compared between two culture conditions. (**C**) Similar to part A except that the mRNA levels of the selected HOX genes were compared between 2D (day 3) and lrECM 3D cultures (day 4) of Hs578T cells. (**D**) Total cell lysates were extracted from 2D (day 3) and lrECM 3D (day 6) cultures of MDA-MB231 cells. The protein levels of HOXA9 were measured using immunoblots. (**E**) The morphology of MDA-MB-231 cells in 2D culture was visualized by immunofluorescence for the intermediate filament vimentin using a vimentin-specific antibody and an Alexa 488-conjugated secondary antibody (pseudocolored in green). The image was captured at 400× magnification. (**F**) The morphology of MDA-MB-231 cells in lrECM 3D culture (day 6) was visualized by staining for filamentous actin using Alexa 488 conjugated phalloidin (pseudocolored in green). The stellate projections were indicated by red arrowheads. In part E and F the nucleus was stained using DAPI (pseudocolored in blue). The image was captured at 200× magnification. When presented, means and standard deviations were obtained from three independent experiments. *, **, and *** indicate a *P* value < 0.05, 0.01, 0.001, respectively. In Figure 1A, *P* values range from < 0.05 to < 0.001. For the sake of presentation, we used the largest *P* value (*) to indicate the statistical difference between 2D culture and each indicated time point of lrECM 3D culture.

The crosstalk between ECM signaling and epigenetic coding might be particularly important to the Claudin-low subtype because its gene expression signature is enriched with a cluster of ~80 genes featuring ECM genes (*e.g.*, laminin) and their receptors (*e.g.*, integrins) [6]. To explore this crosstalk we employed lrECM 3D culture because it yields distinctive gene expression signatures of each intrinsic subtype of breast cancer [21]. We chose the Claudin-low breast cancer cell lines MDA-MB-231 and Hs578T because of their invasive and metastatic competence [6, 25]. We focused on the HOX genes because they are tightly regulated by epigenetic codes and critical to breast cancer [15, 16]. We reasoned that differential expression of the HOX genes between 2D and lrECM 3D cultures would reflect distinct epigenetic coding of the HOX genes between two culture conditions. We observed a robust induction of the selected HOX genes (HOXA9, HOXB7, HOXB13, HOXD10) in lrECM 3D culture over 2D culture of MDA-MB-231 cells and Hs578T cells (Figure 1A–1C). Induction of the HOX genes occurred as early as day 2 and sustained through day 10 (Figure 1A–1C). HOXA9 exhibited the greatest increase in MDA-MB-231 cells, whereas HOXB7 exhibited the greatest increase in Hs578T cells (Figure 1B and 1C). We confirmed increase of the protein levels of HOXA9 in lrECM 3D culture of MDA-MB-231 cells over 2D culture (Figure 1D). However it was noteworthy that the increase of the protein levels of HOXA9 was much smaller than the mRNA levels. This suggested post-transcriptional regulation of HOXA9 expression in lrECM 3D culture. A survey of a recent gene expression profiling (GSE36953) confirmed widespread differential expression of the HOX genes between 2D and lrECM 3D cultures of MDA-MB-231 cells (Supplementary Materials Table S2) [26]. In 2D culture, MDA-MB-231 cells exhibited mesenchymal cell like morphoglogy that featured absence of cell-cell adhesion and robust expression of vimentin, a mesenchymal cell-specific intermediate filament (1E). In lrECM 3D culture MDA-MB-231 cells exhibited stellate projection that is a signature morphology of invasive cancer cells (Figure 1F, indicated by arrowheads) [21].

**Figure 2:**
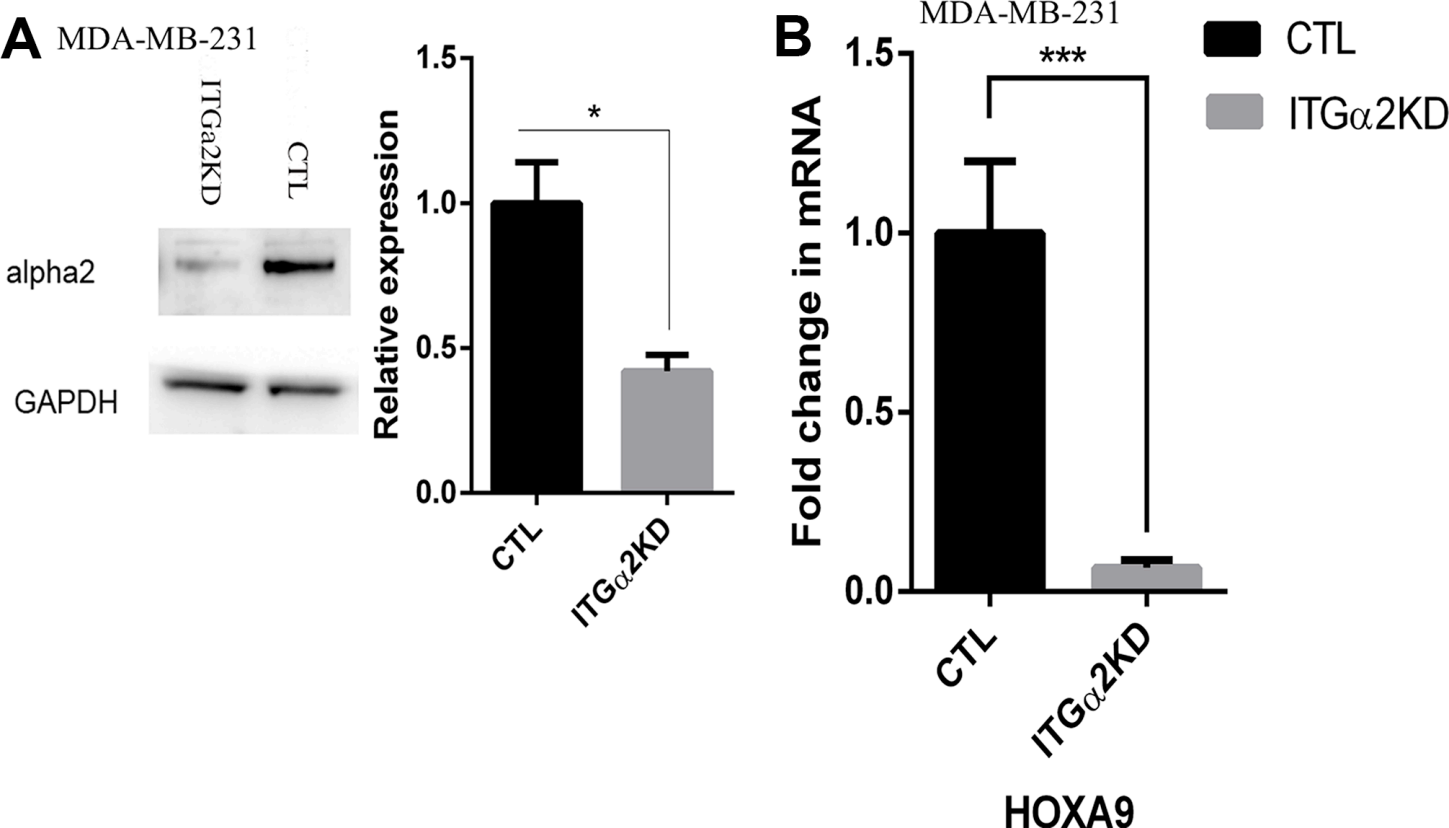
Reduced expression of HOXA9 by inhibition of integrin α2 (**A**) Total cell lysates were extracted from two MDA-MB-231 cells variants that were transduced with lentiviral particles expressing integrin α2-specific Mission shRNA (ITGα2KD) or a matching control shRNA (CTL). The protein levels of integrin α2 were measured using immunoblots. (**B**) Total cell RNA was extracted from the ITGα2KD and CTL variants on day 6 of lrECM 3D culture. The mRNA levels of HOXA9 were measured using qRT-PCR. A fold change of HOXA9 in ITGα2KD cells over CTL cells was obtained by normalizing to the housekeeping gene RPLP0 and setting the values from CTL cells to one. When presented, means and standard deviations were obtained from three independent experiments. * and *** indicate a *P* value < 0.05 and 0.001, respectively.

Among the up-regulated HOX genes in lrECM 3D culture we chose to focus on HOXA9 because of its robust induction in lrECM 3D culture and its critical roles in breast cancer (Figure 1B–1D) [27–29]. To question whether induction of HOXA9 required ECM signaling in lrECM 3D culture we generated an MDA-MB-231 variant in which integrin α2, a major receptor for ECM, was reduced by the stably expressed integrin α2-specific shRNA (ITGα2KD). The protein levels of integrin α2 were substantially reduced in ITGα2KD when compared with a matching control variant (CTL) (Figure 2A). Then we compared the mRNA levels of HOXA9 between lrECM 3D cultures of ITGα2KD and CTL variants. The mRNA levels of HOXA9 in the ITGα2KD variant were reduced to 7% of that in the CTL variant (Figure 2B). Morphogenesis and expression of HOXA9 in the CTL variant were comparable to the parental MDA-MB-231 cells (data not shown).

### Disparate epigenetic regulation of the HOXA9 promoter between 2D and lrECM 3D cultures

**Figure 3:**
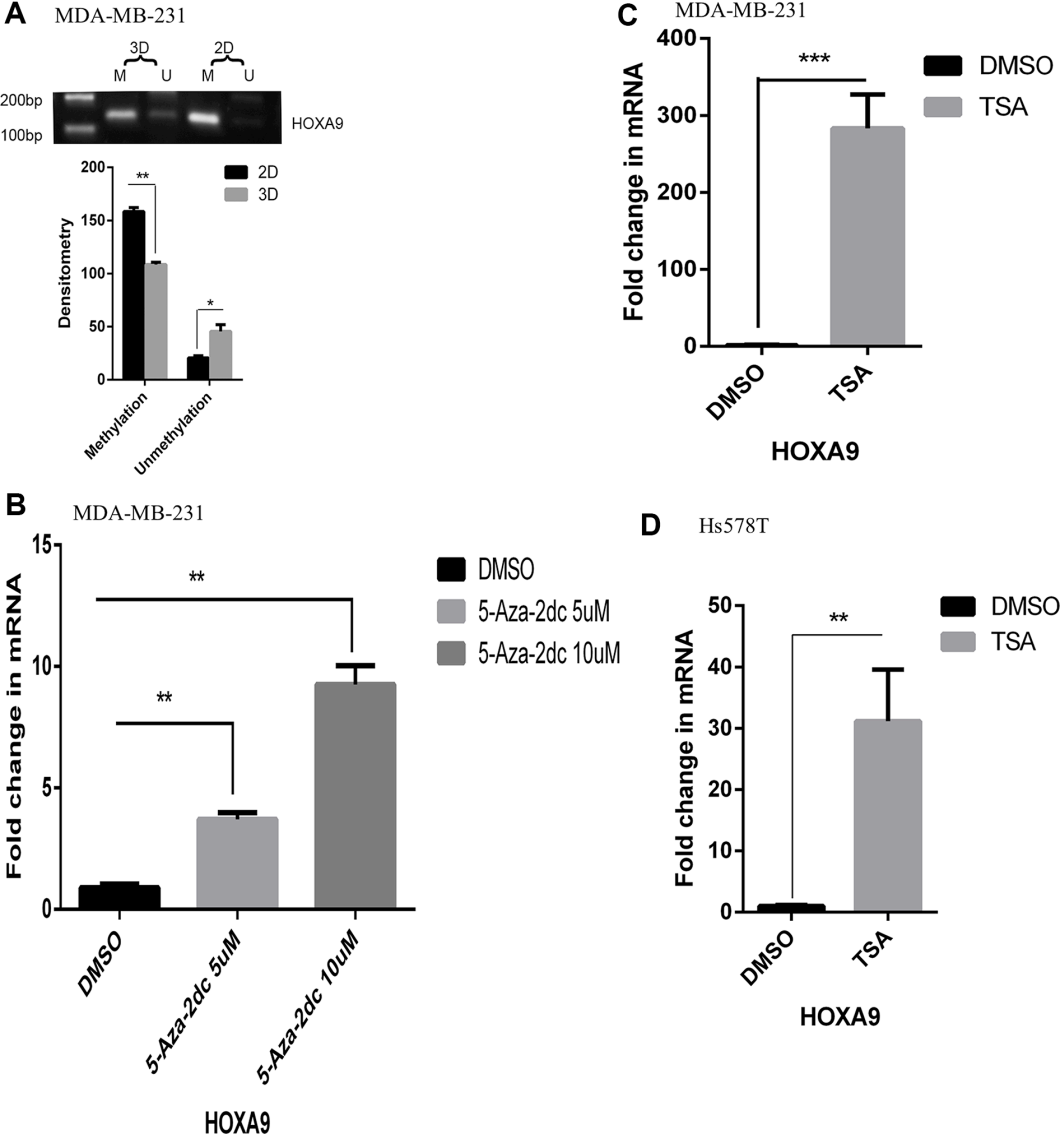
Hypomethylation of the HOXA9 promoter in lrECM 3D culture (**A**) Total cell DNA was extracted from MDA-MB-231 cells in 2D and lrECM 3D cultures on day 6. CpG methylation of the HOXA9 promoter was compared between two culture conditions using bisulfite treatment coupled with methylation-specific PCR. The intensity of each PCR product was quantified by densitometry using NIH Image J. (**B**) Total cell RNA was extracted from 2D culture of MDA-MB-231 cells with exposure to either a DNMT inhibitor, 5-Aza-2dC (5 & 10 μM) or DMSO for 72 hrs. The mRNA levels of HOXA9 were measured using qRT-PCR. A fold change was obtained by normalizing to the housekeeping gene RPLP0 and setting thevalues from the DMSO group to one. (**C**) Similar to part B except that the mRNA levels of HOXA9 were compared between the TSA (500 nM, 72 hrs) treated group and the DMSO treated group. (**D**) Similar to part C except that the mRNA levels of HOXA9 were compared between the TSA (500 nM, 72 hrs) treated group and the DMSO treated group in Hs578T cells. When presented, means and standard deviations were obtained from three independent experiments. *, **, *** indicate a *P* value < 0.05, 0.01, 0.001, respectively.

We postulated that activation of HOXA9 correlated with CpG hypomethylation of the HOXA9 promoter in lrECM 3D culture because HOXA9 is repressed by cytosine hypermethylation [30]. We compared CpG methylation of the HOXA9 promoter between 2D and lrECM 3D cultures of MDA-MB-231 cells using bisulfite treatment and methylation-specific PCR as described elsewhere [31]. We designed the methylation- and unmethylation-specific primers using MethPrimer to measure methylation of two CpG sites within −918 bp to −772 bp upstream of the HOXA9 transcription start site (illustrated in Supplementary Materials Table S1) [32]. The methylation-specific PCR products of the HOXA9 promoter exhibited a 32% decrease in lrECM 3D culture over 2D culture (Figure 3A). In contrast the unmethylation-specific PCR products exhibited a 120% increase (Figure 3A). These results demonstrated an inverse correlation between HOXA9 expression and methylation of the HOXA9 promoter (Figure 1B and Figure 3A). We questioned whether inhibition of CpG methylation was sufficient to activate HOXA9 expression in 2D culture. We treated MDA-MB-231 cells in 2D culture with a DNMT inhibitor, 5-Aza-2dC (5 μM and 10 μM) for 72 hrs. The mRNA levels of HOXA9 were stimulated to 4.1-fold (5 μM) and 10.3-fold (10 μM) increase by 5-Aza-2dC over the vehicle DMSO treated group (Figure 3B). We questioned whether inhibition of HDAC was able to activate HOXA9 expression in 2D culture because DNMT and histone deacetylases (HDAC) commonly work in concert to silence a gene promoter [33]. TSA (500 nM, 72 hrs) induced the mRNA levels of HOXA9 to 282– and 31-fold increase over the DMSO-treated group in MDA-MB-231 and Hs578T cells, respectively (Figure 3C and 3D). These findings suggested that DNMT and HDAC repressed the HOXA9 promoter in 2D culture and such repression was relieved in lrECM 3D culture.

### Requirement of BRD4 for induction of HOXA9 expression in lrECM 3D culture

**Figure 4:**
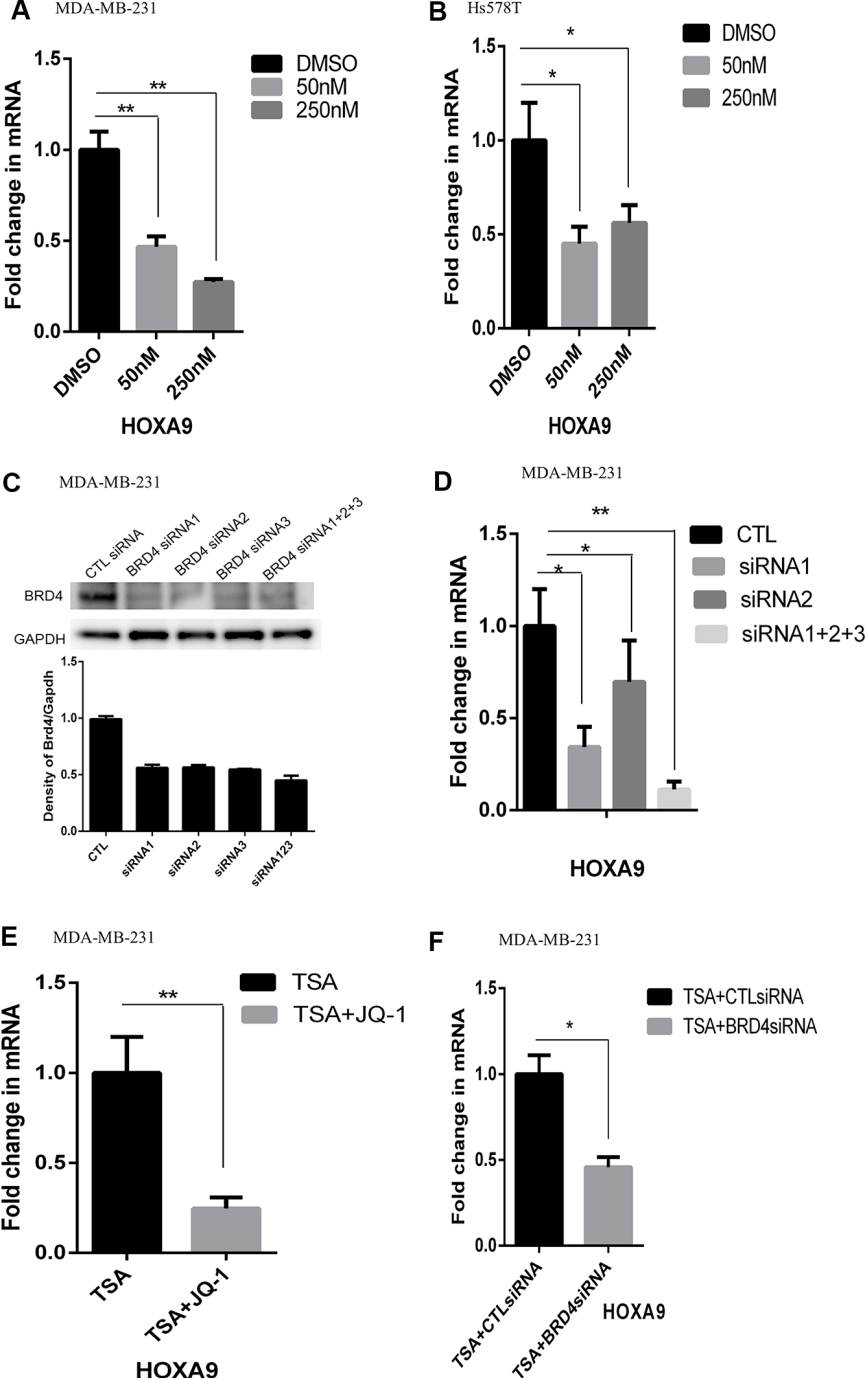
Reduced expression of HOXA9 by inhibition of BRD4 (**A**) Total cell RNA was extracted from lrECM 3D cultures of MDA-MB-231 cells treated with either a BRD4-specific inhibitor JQ1 (50 and 250 nM) or DMSO for 4 days. The mRNA levels of HOXA9 were measured using qRT-PCR. A fold change of the HOXA9 mRNA was obtained by normalizing to the house keeping gene RPLP0 and setting the values from the DMSO treated group to one. (**B**) Similar to part A except that total RNA was extracted from Hs578T cells cultured under the same condition. (**C**) MDA-MB-231 cells were transfected with either BRD4-specific siRNAs (BRD4si, three distinct siRNA labeled as 1–3) or the matching control siRNAs (CTL siRNA). The protein levels of BRD4 were measured using immunoblots and quantified using densitometry. (**D**) MDA-MB-231 cells were transfected with either BRD4-specific siRNAs or the matching control siRNAs (CTL) and seeded in lrECM 3D culture. Total cell RNA was extracted on day 4 in lrECM 3D culture. The mRNA levels of HOXA9 were measured using qRT-PCR. A fold change of the HOXA9 mRNA in the BRD4-specific siRNA groups (siRNA1, siRNA2, siRNA1+2+3) over that in the CTL siRNA (CTL) group was obtained by normalizing to the housekeeping gene RPLP0 and setting the values from the CTL group to one. (**E**) Total cell RNA was extracted from 2D culture of MDA-MB-231 cells treated with either TSA (500 nM) alone or a combination of TSA and JQ1 (250 nM) for 72 hrs. The mRNA levels of HOXA9 were measured using qRT-PCR. A fold change of the HOXA9 mRNA in the TSA and JQ1 treated group over the TSA group was obtained by normalizing to the housekeeping gene RPLP0 and setting the values from the TSA group to one. (**F**) Similar to part E except that the mRNA levels of HOXA9 were measured in 2D culture of MDA-MB-231 cells exposed to TSA (500 nM) with transfection of control siRNA (CTLsiRNA) or the BRD4-specific siRNA (BRD4siRNA, a pool of siRNA1–3). When presented, means and standard deviations were obtained from three independent experiments. * and ** indicate a *P* value < 0.05 and 0.01, respectively.

BRD4 binds the acetylated histones to promote gene expression [17, 18]. We speculated that BRD4 promoted HOXA9 expression in lrECM 3D culture because inhibition of HDAC activated HOXA9 expression in 2D culture (Figure 3C). We treated lrECM 3D culture of MDA-MB-231 and Hs578T cells with a BRD4-specific inhibitor JQ1 for 4 days. JQ1 (50 nM & 250 nM) substantially reduced the mRNA levels of HOXA9 in lrECM 3D culture of both cell lines (Figure 4A and 4B). To confirm requirement of BRD4 for activation of HOXA9 we transfected MDA-MB-231 cells with the BRD4-specific siRNAs or control siRNAs. The protein levels of BRD4 were substantially reduced by the BRD4 siRNAs when compared with the control siRNA group (Figure 4C). The mRNA levels of HOXA9 were substantially reduced by the BRD4 siRNAs (two individual siRNAs and a pool of three siRNAs) when compared with the control siRNA group in lrECM 3D culture (Figure 4D). We postulated that BRD4 was also required for activation of HOXA9 by the HDAC inhibitor TSA in 2D culture. We exposed MDA-MB-231 cells to TSA alone (500 nM) with or without JQ1 or the pooled BRD4siRNAs for 72 hrs. JQ1 (250 nM) and the BRD4-specific siRNAs substantially reduced the mRNA levels of HOXA9 in the presence of TSA, respectively (Figure 4E and 4F).

**Figure 5:**
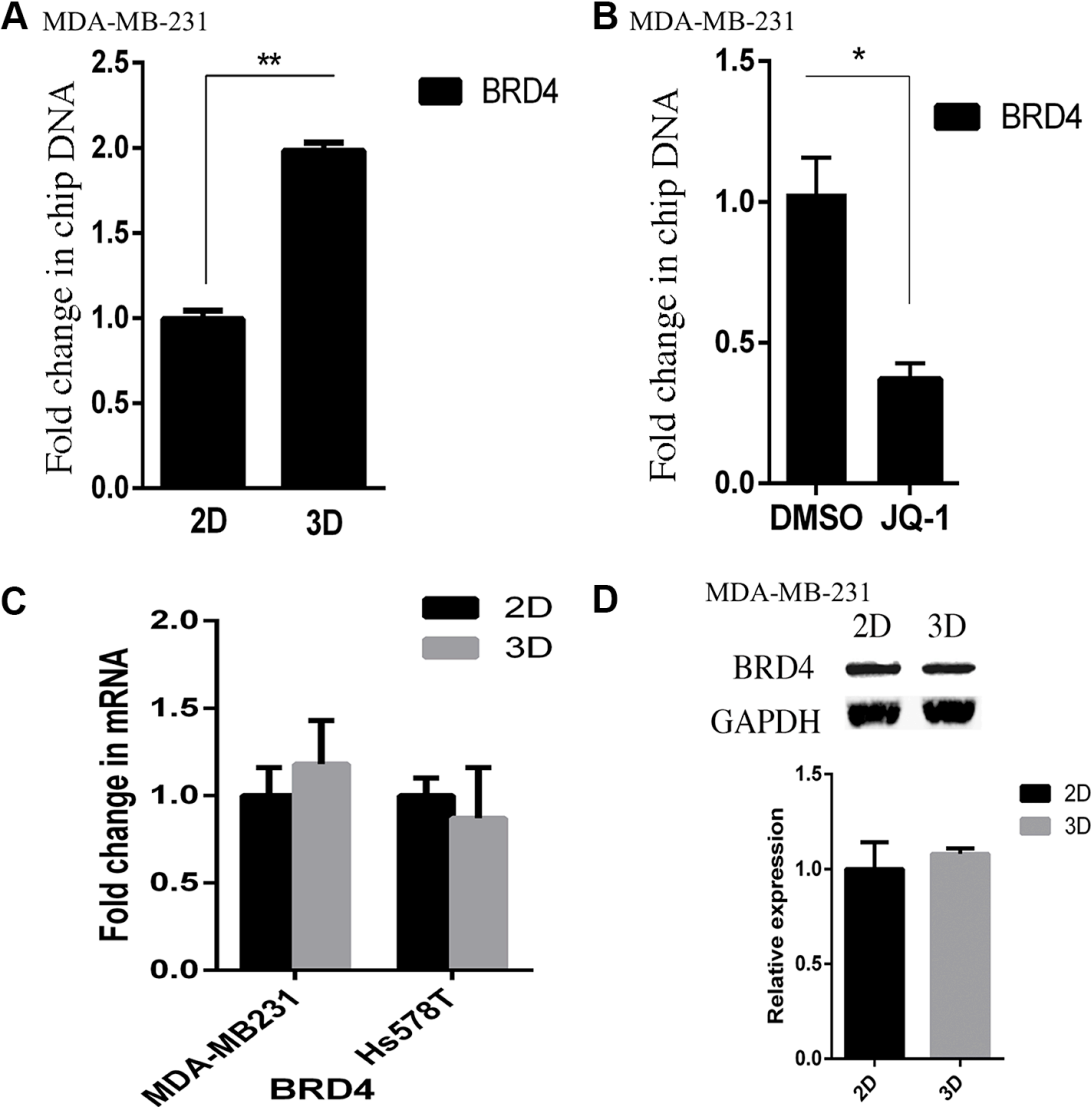
Elevated binding of BRD4 to the HOXA9 promoter in lrECM 3D culture (**A**) ChIP assays were carried out using a BRD4-specific antibody in 2D and lrECM 3D cultures of MDA-MB-231 cells on day 4. The BRD4 bound HOXA9 promoter was measured using qPCR and normalized to their corresponding input. A fold change of the BRD4-bound HOXA9 promoter in lrECM 3D culture over 2D culture was obtained by setting the values from 2D culture to one. (**B**) Similar to part A except that the BRD4-bound HOXA9 promoter was compared between lrECM 3D cultures treated with either JQ1 (250 nM) or DMSO. A fold change of the BRD4-bound HOXA9 promoter in the JQ1 group over the DMSO group was obtained by setting the values from the DMSO group to one. (**C**) Total cell RNA was extracted from MDA-MB-231 and Hs578T cells in 2D and lrECM 3D cultures on day 4. The mRNA levels of BRD4 were compared between 2D and lrECM 3D cultures using qRT-PCR. A fold change of BRD4 mRNA was obtained by normalizing to the housekeeping gene RPLP0 and setting the values from 2D culture to one. (**D**) Similar to part C except that the protein levels of BRD4 were measured using immunoblots. When presented, means and standard deviations were obtained from three independent experiments. * and ** indicate a *P* value < 0.05 and 0.01, respectively.

Then we carried out ChIP assays to compare BRD4 binding to the HOXA9 promoter in 2D and lrECM 3D cultures of MDA-MB-231 cells. We observed a 2-fold increase in the BRD4-bound HOXA9 promoter (−7 to +121 relative to the transcription start site) in lrECM 3D culture over 2D culture (Figure 5A). As a proof of the specificity of our ChIP assays the control antibody bound HOXA9 promoter was below 10% of the BRD4-bound HOXA9 promoter (data not shown). We questioned whether JQ1 disrupted BRD4 binding to the HOXA9 promoter. JQ1 (250 nM) substantially reduced the BRD4 bound HOXA9 promoter to 37% of that in the DMSO treated group (Figure 5B). Meanwhile the mRNA and protein levels of BRD4 exhibited no difference between two culture conditions of MDA-MB-231 cells (Figure 5C and 5D). These data indicated that BRD4 promoted HOXA9 expression in lrECM 3D culture via increased binding to the HOXA9 promoter.

## DISCUSSION

Herein we demonstrated that HOXA9 expression was induced in lrECM 3D culture of the Claudin-low breast cancer cells over conventional 2D culture and such induction was associated with epigenetic activation of the HOXA9 promoter.

In terms of biophysical properties lrECM 3D culture resembles *in vivo* condition better than conventional 2D culture [34]. Our findings indicate CpG hypomethylation and increased BRD4 binding in the HOXA9 promoter in lrECM 3D culture when compared with 2D culture (Figures 3 and 5). Hypomethylation and BRD4 binding are critical to activation of HOXA9 by ECM because: 1) inhibition of BRD4 abrogated activation of HOXA9 expression in lrECM 3D culture; 2) inhibition of BRD4 abrogated activation of HOXA9 expression by an HDAC inhibitor in 2D culture; 3) inhibition of CpG methylation activated HOXA9 expression in 2D culture (Figures 3 and 4). CpG hypomethylation in lrECM 3D culture appears to be cellular and gene context dependent because the antioxidant enzyme EcSOD gene is activated via CpG hypomethylation only in lrECM 3D culture of normal mammary epithelial cells, but not breast cancer cells [24]. Moreover GSK343, an inhibitor of PRC2, is reported to induce cell death of ovarian cancer cells only in lrECM 3D culture, but not 2D culture [35]. Thus prognostic values of gene expression profiles and drug responses obtained from lrECM 3D cultures can potentially provide guidance to treatment of breast cancer [22, 35, 36].

Tension force is implied to regulate expression of the HOX genes [37]. HOXA9 expression is repressed by increased stiffness in the Luminal breast cancer cells via miR-18's targeting of HOXA9 [28]. Induction of HOXA9 in lrECM 3D culture of the Claudin-low cells is consistent with this report because lrECM is much less stiff than the plastic substratum in conventional 2D culture [34]. In the Luminal breast cancer cells HOXA9 suppresses tumor progression via activation of PTEN expression [27, 28]. However a positive correlation between HOXA9 induction and invasive growth in the Claudin-low breast cells apparently contradicts its reported tumor suppressive activity (Figure 1). In the Claudin-low breast cancer cells we observed a slight decrease of PTEN expression in lrECM 3D culture over 2D culture (Supplementary Materials Figure S1). We speculate that HOXA9's tumor suppressive activity is neutralized by other concurrently induced tumor-promoting HOX genes in lrECM 3D culture of the Claudin-low breast cancer cells. One plausible suspect is the lncRNA HOX transcript antisense RNA (HOTAIR) that is transcribed from an intergenic locus between HOXC11 and HOXC12 [38, 39]. HOTAIR reprograms chromatin stage and gene expression to promote invasion and metastasis in breast cancer [40, 41]. Intriguingly HOTAIR is concurrently induced in lrECM 3D culture of the Claudin-low breast cancer cells (unpublished observations). HOTAIR has been reported to repress PTEN expression in laryngeal squamous cell carcinoma [42]. We postulate that induction of HOTAIR and the consequent repression of PTEN by HOTAIR may neutralize activation of PTEN by HOXA9 in lrECM 3D culture.

The Claudin-low subtype is enriched with gene expression signatures that are linked to the ECM genes and their receptors integrins [6, 11]. In the Claudin-low MDA-MB-231 and Hs578T cells a large number of the HOX genes are nearly silenced in 2D culture, but robustly induced in lrECM 3D culture relative to the Luminal MCF-7 and T47D cells (Figure 1, Supplementary Materials Table S2). We demonstrated a panel of 29 miRNAs that are differentially expressed between the Claudin-low (MDA-MB-231) and Luminal A (MCF-7) breast cancer cells only in lrECM 3D culture [43]. These results imply distinct cell responses to ECM among the intrinsic subtypes of breast cancer. This difference depends on ECM signaling because inhibition of integrin α2, a receptor of collagen, reduced HOXA9 expression in lrECM 3D culture of MDA-MB-231 cells (Figure 2). Interference of other integrin receptors, such as integrin α6 and α7 (receptors for laminin), is still needed to determine the role of each individual ECM in regulation of HOXA9 expression in lrECM 3D culture [44–46].

In summary we demonstrate that HOXA9 expression is activated by binding of BRD4 to the CpG hypomethylated HOXA9 promoter in the Claudin-low breast cancer cells attached to ECM. Our findings suggest a novel and pivotal crosstalk between ECM and the Claudin-low breast cancer cells.

## MATERIALS AND METHODS

### Reagents and plasmids

Matrigel and Cell Recovery Solution for Matrigel-based cell culture were purchased from Corning (Bedford, MA). 5-Aza-2′-deoxycytidine (5-Aza-2dC), a DNMT inhibitor, was purchased from BioVision (Milpitas, CA). Trichostatin (TSA), a HDAC inhibitor, was purchased from Cayman Chemical (Ann Arbor, MI). JQ1, a BRD4-specific inhibitor, is provided by Dr. James Bradner at Dana-Farber Cancer Institute [18]. The HOXA9-specific antibody was purchased from Thermo (Rockford, IL). The GAPDH-specific antibody was purchased from Novus Biologicals (Littleton, CO). The BRD4-specific antibody was purchased from Cell Signaling (Danvers MA).

### Cell culture

The Claudin-low human breast cancer cell lines (MDA-MB-231 and Hs578T) were purchased from ATCC and cultured in DMEM as previously described [47]. At 24 hrs after seeding, the cells were exposed to the indicated treatments (5-Aza-2dC or TSA for 72 hrs).

### lrECM 3D organotypic culture

Overlay lrECM 3D culture was carried out as previously described [48]. Briefy, MDA-MB-231 and Hs578T cells were seeded at 2 × 10^5^ cells/well in a 6-well cell culture plate that was coated with Matrigel. DMEM culture medium was supplemented with 4% of Matrigel and replaced every two days. The morphology of cell clusters was monitored for 12 days and recorded using an inverse phase contrast microscope equipped with a digital camera.

### Assessment of morphology

The morphology of MDA-MB-231 cells in 2D culture was visualized by immunofluorescence for vimentin using a vimentin-specific antibody (V6630, Sigma, St. Louis, MO). Briefly, MDA-MB-231 cells were cultured in eight-well chamber slides for 3 days. An Alexa 488 conjugated secondary antibody (1:1,500 dilution) was used to detect the primary antibody. The digital images were captured using Nikon Eclipse 80i along with the accompanying program IPLab (Nikon, Melville, NY, USA). The morphology of MDA-MB-231 cells in lrECM 3D culture was visualized by staining for filamentous actin using Alexa 488 conjugated phalloidin (Invitrogen, Carlsbad CA) followed by confocal fluorescent microscopy on a Bio-Rad Radiance 2100 system (Hercules, CA) [49]. The nucleus was stained using DAPI.

### Retroviral transduction

The integrin α2-specific Mission shRNA lentivrial transduction particles and its matching control were purchased from Sigma (St. Louis MO). Retroviral transduction was carried out as previously described [50]. MDA-MB-231 cells were infected with the retroviruses at an MOI of 0.5. The stably transduced MDA-MB-231 cells were selected using puromycin.

### Transient transfection and RNA interference

Sigma (St. Louis MO) provided the human BRD4-specific Mission siRNAs (BRD4siRNA) with a Sigma ID SIHK0192, SIHK0193, and SIHK0194 and the control Mission siRNA. The siRNAs were transfected at 60 nM into MDA-MB-231 cells using the reverse transfection protocol with RNAiMAX per the provider's instructions (Invitrogen, Carlsbad, CA). Total RNA and protein were extracted at day 3 in 2D culture and day 4 in lrECM 3D culture.

### RNA extraction and analysis of mRNA expression

Total cell RNA was extracted using TRIzol (Invitrogen) from 2D and lrECM 3D cultures at the indicated time points as previously described [51]. Quantitative RT-PCR (qRT-PCR) was carried out to determine the mRNA levels of the genes of interest. Each transcript was normalized to a house keeping gene ribosomal protein large P0 (RPLP0). A fold change of each transcript was obtained by setting the values from the control group to one.

### Immunoblot

Total cell lysates were extracted from MDA-MB-231 cells exposed to the indicated treatments using 1 x Laemmli buffer. In lrECM 3D culture MDA-MB-231 cells were separated from Matrigel using BD cell recover solution as previously described [52]. Immunobloting was used to measure the protein levels of HOXA9, integrin α2, BRD4, a-tubulin, and GAPDH [53]. Each immunoblot was quantified by densitometry using NIH Image J. A fold change of each protein of interest was obtained by normalizing to its corresponding loading control GAPDH or tubulin and setting the values from the control groups to one.

### Bisulfite modification and methylation-specific PCR

Cytosine methylation within the CpG island in the HOXA9 promoter was measured using bisulfite treatment coupled with methylation-specific PCR as described elsewhere [31]. Briefly genomic DNA was extracted from 2D and lrECM 3D cultures of MDA-MB-231 using PureLink Genomic DNA (Invitrogen, Carlsbad CA). Each genomic DNA sample was bisulfite-modified using EZ DNA Methylation-Lightning kit (Zymo Research, Irvine CA). CpG methylation in the HOXA9 promoter was measured using methylation-specific PCR. The methylated- and unmethylated-specific primers were designed using MethPrimer (http://www.urogene.org/cgi-bin/methprimer/methprimer.cgi) [32]. The positions of the methylation sites were illustrated in Supplementary Materials Table S1. The PCR products were electrophoresed and the intensity of each PCR product band was quantified using NIH Image J and compared between 2D and lrECM 3D cultures.

### Chromatin immunoprecipitation

Chromatin immunoprecipitation (ChIP) was performed as we previously described with minor modifications [54]. EZ ChIP Kit (Cat #17–371) and a ChIP grade BRD4-specific antibody were purchased from Millipore (Darmstadt, Germany). Sheared chromatin prepared from roughly 1 × 10^7^ cells was immunoprecipitated with a BRD4-specific antibody or a negative control antibody. Two μl of each ChIP DNA sample was used for quantitative PCR. The sequences of the primers specific for the human HOXA9 promoter were provided in Supplementary Materials Table S1. The input of the HOXA9 promoter in each DNA sample was also measured using qPCR. The ratios of the immunoprecipitated HOXA9 promoter versus its corresponding input were compared between the selected two groups. A fold change of the immunoprecipitated HOXA9 promoter was established by setting the values from control group (2D culture or lrECM 3D culture without any treatment) to one.

### Statistical analysis

Statistical significance between any two selected groups was determined using the unpaired two-tailed Student's *t*-test (Prizm Version 5). A *P* value smaller than 0.05 was considered significant.

## SUPPLEMENTARY MATERIALS FIGURES AND TABLES




