# Supplementary material for: Induction of HOXA9 expression in three-dimensional organotypic culture of the Claudin-low breast cancer cells
Source: Oncotarget. 2016 Jul 8;7(32):51503–14. doi: 10.18632/oncotarget.10491 (PMC5239492; doi:10.18632/oncotarget.10491)
Supplement: Supplementary file 1 [file oncotarget-07-51503-s001.pdf]

Induction of HOXA9 expression in three-dimensional organotypic culture of the Claudin-low breast cancer cells

Supplementary Materials

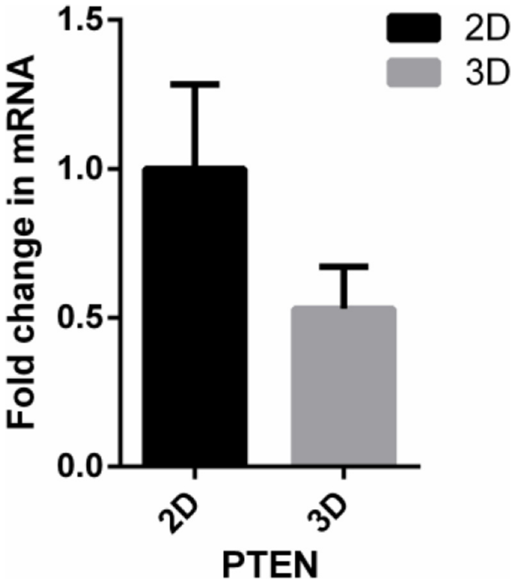

Supplementary Figure S1: Expression of PTEN in 2D and lrECM 3D culture of MDA-MB-231 cells. Total cell RNA was extracted from MDA-MB-231 cells in 2D and lrECM 3D cultures on day 6. The mRNA levels PTEN were compared between 2D and lrECM 3D cultures. A fold change of PTEN mRNA in lrECM 3D culture over that in 2D culture was obtained by normalizing to the housekeeping gene RPLP0 and setting the values from 2D culture to one. Means and standard deviations were obtained from three independent experiments.

Supplementary Table S1: Sequences of the primers

Names of the Primers	Sequences of the Primers
hHOXA9_F_+549	5′-GTGGTTCTCCTCCAGTTGATAG-3′
hHOXA9_R_+672	5′-AGTTGGCTGCTGGGTTATT-3′
hHOXA9_M_F_–918	5′-TTAATTAGGTTTTTAAAATTTTCGT-3′
hHOXA9_M_R_–774	5′-TATAAATCCCCACAACTACCCG-3′
hHOXA9_U_F_–918	5′-TTAATTAGGTTTTTAAAATTTTTGT-3′
hHOXA9_U_R_–772	5′-AATATAAATCCCCACAACTACCCA-3′
hHOXA9_F_–7	5′-TGCCACCAAGTTGTTACATGA-3′
hHOXA9_R_+121	5′-AGGAACGAGTCCACGTAGTAG-3′
hRPLP0_F_+197	5′-CGACCTGGAAGTCCAACTAC-3′
hRPLP0_F_+305	5′-ATCTGCTGCATCTGCTTG-3′
hPTEN_F_+1313	5′-CCCACCACAGCTAGAACTTATC-3′
hPTEN_R_+1422	5′-TCGTCCCTTTCCAGCTTTAC-3′
hHOXB7_F_+71	5′-AACGTCCCTGCCTACAAATC-3
hHOXB7_R_+212	5′-GGAAGCAAACGCACAAGAAG-3′
hHOXB13_F_+261	5′-GGGTTCAAGGAAGATGGAGATG-3′
hHOXB13_R_+363	5′-CATTTAGGTCTTGTCCGTGTGA-3′
hHOXD10_F_+1654	5′-GTCCTTGGTGAGATGGGATATTG-3′
hHOXD10_R_+1749	5′-GGACAGGTTGCTGTTGTACTT-3′

Human HOXA9 (GenBanck Accession #: NM_152739); Human RPLP0 (GenBanck Accession #: NM_001002); Human PTEN (GenBanck Accession #: NM_000314); Human HOXB7 (GenBanck Accession #: NM_004502); Human HOXB13 (GenBanck Accession #: NM_006352), Human HOXD10 (GenBanck Accession #: NM_002148). The numbers in each primer indicates the 5′ end position of each primer relative to the corresponding transcription start site. A minus sign preceding the numbers indicates upstream of the transcription start site and a plus sign indicates downstream of the transcription start site. F strands for forward primers and R stands for reverse primers. M stands for the methylation-specific primer and U stands for the unmethylation-specific primer. The CpG methylation sites are highlighted in the HOXA9 methylation/unmethylation-specific primers.

Supplementary Table S2: Differential expression of the HOX genes between 2D and lrECM 3D cultures of MDA-MB-231 cells. See Supplementary_Table_S2
