# Supplementary material for: Induction of HOXA9 expression in three-dimensional organotypic culture of the Claudin-low breast cancer cells
Source: Oncotarget. 2016 Jul 8;7(32):51503–14. doi: 10.18632/oncotarget.10491 (PMC5239492; doi:10.18632/oncotarget.10491)
Supplement: Supplementary file 2 [file oncotarget-07-51503-s002.docx]

Supplementary Table S2: Differential expression of the HOX genes between 2D and lrECM 3D cultures of MDA-MB-231 cells

| **Probe ID** | **2D** | **3D** | **Fold** |
| --- | --- | --- | --- |
| 244512_at: HOXB-AS3 | 0.69 | 5.37 | 25.63 |
| 238808_at: HOXA13 | 1.16 | 4.87 | 13.09 |
| 1557050_at: HOTAIRM1 | 0 | 3.68 | 12.82 |
| 243811_at: HOXB1 | 0.32 | 3.93 | 12.21 |
| 239153_at: HOTAIR | 0.09 | 3.54 | 10.93 |
| 231786_at: HOXA13 | 0 | 3.21 | 9.25 |
| 205365_at: HOXB6 | 1.65 | 4.74 | 8.51 |
| 229493_at: HOXD-AS2 | 2.12 | 5.17 | 8.28 |
| 1564070_s_at: HOTTIP | 2.82 | 5.78 | 7.78 |
| 231906_at: HOXD8 | 2.18 | 4.62 | 5.43 |
| 205605_at: HOXD9 | 1.98 | 4.3 | 4.99 |
| 208414_s_at: HOXB3 | 3.73 | 6.02 | 4.89 |
| 205366_s_at: HOXB6 | 2.22 | 4.49 | 4.82 |
| 214457_at: HOXA2 | 2.19 | 4.4 | 4.63 |
| 239182_at: HOXD-AS1 | 2.78 | 4.51 | 3.32 |
| 1557051_s_at: HOTAIRM1 | 2.76 | 4.41 | 3.14 |
| 221411_at: HOXD12 | 0.37 | 1.72 | 2.55 |
| 207398_at: HOXD13 | 2.91 | 4.26 | 2.55 |
| 231767_at: HOXB4 | 4.15 | 5.45 | 2.46 |
| 213823_at: HOXA11 | 3.11 | 4.39 | 2.43 |
| 204778_x_at: HOXB7 | 4.06 | 5.28 | 2.33 |
| 236893_at: HOXB-AS3 | 0.29 | 1.46 | 2.25 |
| 205600_x_at: HOXB5 | 5.49 | 6.62 | 2.19 |
| 1564069_at: HOTTIP | 5.26 | 6.36 | 2.14 |
| 236681_at: HOXD13 | 4.23 | 5.33 | 2.14 |
| 205975_s_at: HOXD1 | 8.59 | 9.64 | 2.07 |
| 205974_at: HOXD1 | 2.08 | 3.12 | 2.06 |
| 213147_at: HOXA10 | 5.75 | 4.72 | 0.49 |
| 1552337_s_at: HOXD4 | 7.14 | 5.93 | 0.43 |
| 209905_at: HOXA10-HOXA9 | 1.58 | 0.34 | 0.42 |
| 235521_at: HOXA3 | 4.35 | 3.04 | 0.40 |
| 206289_at: HOXA4 | 5.28 | 3.83 | 0.37 |
| 207373_at: HOXD10 | 2.66 | 1.15 | 0.35 |
| 206739_at: HOXC5 | 7.18 | 5.66 | 0.35 |
| 239915_at: HOXA-AS3 | 4.91 | 3.21 | 0.31 |
| 214639_s_at: HOXA1 | 4.66 | 2.94 | 0.30 |
| 218959_at: HOXC10 | 4.58 | 2.86 | 0.30 |
| 208557_at: HOXA6 | 4.75 | 2.97 | 0.29 |
| 213150_at: HOXA10 | 3.64 | 1.78 | 0.28 |
| 214604_at: HOXD11 | 4.2 | 1.96 | 0.21 |
| 206602_s_at: HOXD3 | 3.49 | 1.17 | 0.20 |
| 230845_at: HOXB-AS5 | 5.73 | 3.37 | 0.19 |
| 213844_at: HOXA5 | 4.18 | 1.78 | 0.19 |
| 240151_at: HOXB-AS3 | 3.39 | 0.85 | 0.17 |
| 230080_at: HOXA-AS2 | 5.67 | 2.97 | 0.15 |
| 205601_s_at: HOXB5 | 6.16 | 3.16 | 0.13 |
| 236892_s_at: HOXB-AS3 | 4.7 | 0.53 | 0.06 |
| 206847_s_at: HOXA7 | 5.52 | 1.34 | 0.06 |
| The log2 transformed counts of the probe sets that recognize the HOX genes were extracted from an AffyMetrix array GSE36953 that compared gene expression profiles between 2D and lrECM 3D cultures of MDA-MB-231 cells. The list included the HOX genes that were differentially expressed at or above two fold between 2D and lrECM 3D cultures. | | | |
